# Evaluating National Circular Economy Practices in the UK: Setting a Strategic Agenda for a Nation-Wide Roadmap

**DOI:** 10.1007/s43615-025-00669-2

**Published:** 2025-08-08

**Authors:** Halidu Abu-Bakar, Fiona Charnley

**Affiliations:** Faculty of Environment, Science and Economy, University of Exeter Business School, Rennes Dr, Exeter, EX4 4PU England

**Keywords:** Circular economy roadmaps, Natural Language Processing (NLP), Regional disparities, CE implementation strategies, Policy document analysis, Sectoral sustainability practices

## Abstract

This research presents a comprehensive analysis of Circular Economy (CE) practices across the United Kingdom, using advanced Natural Language Processing (NLP) techniques, specifically Named Entity Recognition (NER), chosen for its reproducible, large-scale extraction of locations, sectors and 9R actions from 1.2 million words of policy text. The study examines 36 key documents, comprising roadmaps, policy statements, and sectoral reports, to categorise CE activities such as recycling, reduction, reuse, and recovery. The dataset includes 22,589 distinct entries, covering 52 locations, 34 industrial sectors, and 109 stakeholder categories. Notably, recycling emerges as the most dominant activity, representing 42.8% of all practices, which suggests an over-reliance on waste management solutions rather than upstream interventions like reduction and remanufacturing. Analysis shows that Construction & Demolition (19.8%) and Food & Beverage (13.7%) account for most initiatives, while the Digital, Electronics and Aviation sectors together contribute barely 1%. Local authorities lead 18% of actions, yet trade organisations add less than 1%. Regional priorities also differ: Wales directs 46% of activity to waste management, whereas Scotland and Northern Ireland devote 44% and 53% respectively to decarbonisation and resource-efficiency measures. These disparities reveal structural asymmetries in investment and skills and therefore justify a devolved-yet-co-ordinated policy mechanism, similar to the UK Industrial Strategy Council, that can harmonise regional targets while preserving local strengths. This research offers critical insights into sectoral and geographic patterns, allowing policymakers to prioritise gaps in existing CE initiatives. Although recycling diverts material from landfill, our results indicate that this downstream concentration yields lower value-retention and slower decarbonisation than upstream actions (e.g. design-for-reuse or remanufacturing), signalling an urgent need to rebalance UK policy towards the upper tiers of the 9R hierarchy. Notably, our findings reveal a fragmented approach to CE in the UK– with regions pursuing different priorities and a heavy reliance on recycling– underscoring the need for a unified national CE roadmap. We therefore recommend the development of an integrated UK-wide CE strategy that incentivises upstream practices (e.g. reduction and reuse) and harmonises regional efforts to achieve broader circular economy goals.

## Highlights

In-depth analysis of UK Circular Economy practices, uncovering regional and sectoral disparities.

Innovative use of NLP and NER for analysing CE strategies, providing a novel methodological approach.

Emphasises the need to prioritise upstream practices like reduction, reuse, and remanufacturing.

Advocates for a unified UK-wide CE roadmap that integrates regional strengths and addresses sectoral gaps.

Highlights the critical role of broadening stakeholder engagement to advance CE initiatives effectively.

## Introduction

The origins of Circular Economy (CE) in the United Kingdom can be traced back to the growing recognition of sustainable resource management within national policy, which has evolved significantly from its early focus on environmental conservation to its current role in enhancing economic resilience and sustainability. The pivotal moment came with the Pearce Report in 1989, which emphasised the economic benefits of sustainable resource use and laid the groundwork for the integration of CE principles into UK policy frameworks [1]. Over the years, this foundation has been further strengthened by a series of governmental initiatives aimed at embedding CE principles into national planning [2, 3]. The United Kingdom’s early policy trajectory concentrated on recycling, successively shaped by the 1994 Sustainable Development Strategy, the 2008 transposition of the EU Waste Framework Directive and, most recently, the 2018 Resources & Waste Strategy for England; each document tightened material-recovery targets and entrenched today’s dominance of downstream actions [3–5].

At the heart of CE practices are the hierarchical strategies that range from refusing unnecessary resources, reducing inputs, and reusing and repairing products, to the downstream actions of recycling and recovering materials [6, 7]. These nine actions are adopted by DEFRA as the official monitoring framework for England’s CE transition [8] and align with UNEP’s indicator family [9], facilitating benchmarking against other nations; hence they provide the most policy-relevant lens for this study.

To interpret the hierarchy, we draw on three complementary models. First, the Value-Hill illustrates how interventions on the uphill slope, refuse, reduce, reuse, retain far greater economic and material value than those on the downhill side, such as recycling or energy recovery [10]. Second, the Ellen MacArthur Foundation’s three principles, eliminate waste and pollution, circulate products and materials, regenerate nature, supply normative goals against which UK performance can be gauged [11]. Third, the ReSOLVE framework (Regenerate, Share, Optimise, Loop, Virtualise, Exchange) translates those principles into practical levers, for example, product-as-a-service and material substitution, that shift activity towards the high-value zone of the Value-Hill [12].

These practices aim to maximise value retention throughout a product’s lifecycle, with a clear preference for upstream interventions such as refusal and reduction, which ensure the highest value retention by minimising waste at the design stage [10]. While recovery plays a role in the end-of-life phase, particularly in terms of energy recovery from waste, it is considered less desirable within the hierarchy because it focuses on extracting residual value from materials that are no longer recyclable, typically through incineration or energy recovery processes. This reinforces the need for a strategic focus on upstream practices that maintain material value and prevent depletion. Alongside these techno-economic interventions, bio-based strategies like cascading are equally vital. Cascading, for instance, supports the extended use of biological materials through multiple stages, ensuring that both technical and biological cycles contribute to the broader goals of a CE [13].

In alignment with broader sustainability efforts, the UK adopted the Waste Hierarchy under the Waste (England and Wales) Regulations 2011. This hierarchy prioritises environmental sustainability by advocating waste prevention as the primary objective, subsequently followed by preparation for reuse, recycling, recovery, and ultimately, disposal, the least favoured option. The hierarchical prioritisation explicitly underscores the reduction of material throughput during production, mandating efficiency in resource use and energy consumption as foundational elements of the manufacturing process. This strategic shift from conventional waste disposal to sustainable practices underscores the UK’s commitment to resource efficiency and waste reduction, pivotal in advancing the national CE agenda [14].

UK’s strategies have further evolved significantly, influenced by broader European regulatory frameworks such as the EU directives, which have mandated systemic changes in waste management and resource efficiency. This shift from traditional waste management to a more circular approach has been primarily driven by European policy, notably the Waste Framework Directive, which established a foundation for comprehensive waste prevention plans [5, 15]. A notable feature of these evolving strategies is the incorporation of remanufacturing processes, particularly in Scotland and Wales. These processes involve the systematic disassembly and reassembly of products, which serve to extend the lifecycle of goods, there by integrating sustainability directly into the industrial fabric of these regions. The implementation of these policies exhibits pronounced regional variations, with Scotland and Wales making more definitive strides through initiatives such as the Scottish Institute of Remanufacture and the ‘One Planet Living’ commitment, respectively [16, 17]. In contrast,England and Northern Ireland have seen a more business-centric approach with initiatives like the Waste & Resources Action Programme (WRAP’s) Electronic and Electricals Sustainability Action Plan [18].

Local initiatives also play a pivotal role in the UK’s CE strategy, with targeted approaches such as London and Glasgow’s Route Maps and the West Midlands’ focus on leveraging industrial strengths in the automotive and construction sectors. London’s CE Route Map illustrates an ambitious local endeavour, projecting annual economic benefits of at least £7 billion by 2036 from concentrated efforts in sectors such as the built environment, food, textiles, electricals, and plastics. These initiatives demonstrate a clear operationalisation of the ‘reuse’ principle, where products and materials are deliberately reintroduced into the economy with minimal reprocessing. Such approaches are not only resource-efficient but also align with the CE’s strategic goal of reducing dependency on virgin materials. These efforts are complemented by projects in cities like Brighton & Hove, where community-focused strategies support local enterprises in integrating circular principles effectively, enhancing local resilience while fostering sustainable community practices [19–22]. 

The development of reverse supply chains and recycling capacities, particularly for Rare Earth Elements (REE) utilised in critical technologies such as wind turbines and electric vehicles, underscores the necessity of integrating CE models into the UK’s broader environmental strategy. The expansion of recycling infrastructures is pivotal, as it facilitates the reprocessing of materials at the end of their initial life cycle, ensuring their reintegration into the production process and the reduction of virgin material demand. Current projects aim to address these gaps by scaling up recycling operations and establishing comprehensive end-of-life treatment infrastructures [23].

Nationally, the integration of policy and innovation underpins the UK’s CE strategy, with ambitious environmental targets such as recycling 65% of municipal waste by 2035 and significantly reducing landfill use [24]. Central to achieving these targets is the systematic incorporation of repair and refurbishment strategies within the product lifecycle, ensuring that products are maintained and upgraded to extend their utility, thereby mitigating the environmental impacts associated with the production of new goods [24]. Interdisciplinary centres contribute to this strategy by developing new materials and processes that extend product lifecycles and reduce environmental impacts, while educational and consumer engagement initiatives are shifting consumer behaviour towards more sustainable practices [24–27].

The potential transformation of the UK economy under a fully integrated CE model is substantial. Comparing material flows from 2010 to projections for 2020, the anticipated changes include a reduction of 30 million tonnes in material inputs, a 20% decrease in waste generation, and an increase of 20 million tonnes of recycled materials back into the economy [24, 27]. Economically, the shift towards a CE is anticipated to offer substantial benefits, potentially saving UK businesses up to £23 billion annually through improved resource efficiency​ [27]​. This economic incentive is vital for encouraging businesses to adopt circular practices and invest in sustainable technologies.

Despite these advancements, significant challenges remain in harmonising the diverse regional strategies to form a cohesive UK-wide CE framework. The complexities of aligning varied regional approaches with national objectives necessitate comprehensive research and collaborative policymaking. A critical aspect of this harmonisation will be the standardisation of recovery processes across regions, ensuring that waste materials are consistently converted into valuable resources. The lack of existing strategic parameters to inform the development of a unified national roadmap presents a critical gap in the current CE policy landscape.

Abu-Bakar & Charnley [28] introduce a theoretical framework for CE roadmapping, addressing fragmented strategies. The proposed phases of the framework: Scoping and Goal Setting, Baseline Assessment and Processes, Strategy Development, and Implementation and Monitoring, provide a structured approach to CE planning, enhancing coherence and strategic alignment in CE initiatives. Additionally, Abu-Bakar et al. [29] present a typological framework for categorising CE Roadmaps (CERMs) across various governance levels. This framework, based on a comprehensive review of multiple CE documents using natural language processing (NLP) and topic modelling, categorises CERMs into five types: National, Municipal, Regional, Baseline, and Sectoral. The framework highlights the dominant priority of sustainable waste management across different types and underscores the need for standardising CERM approaches to enhance comparability and cross-sectoral learning.

Notwithstanding multiple regional reports, no peer-reviewed study to date compares all four UK nations across the full 9R spectrum using a uniform, replicable analytic method, an evidence gap this paper addresses. This paper seeks to critically evaluate the effectiveness and regional disparities in the implementation of core CE practices, the “Rs” (refuse, reduce, reuse, repair, refurbish, remanufacture, repurpose, recycle, and recover), across the UK, to derive actionable insights to inform the development of a comprehensive framework that fosters multi-stakeholder collaboration between the private and public sectors, ultimately contributing to the formulation of a unified, UK-wide CE roadmap. As the first study of its kind to comprehensively assess the national implementation of these practices across multiple sectors and regions, this analysis will focus on how these practices are influenced by sector-specific requirements and stakeholder interactions, identifying operational efficiencies, policy gaps, and regional variations in their application. The key objectives to achieve this aim include:

A. **Conduct a comprehensive literature review on UK-specific CE**
  Conduct a comprehensive literature review on UK-specific CE publications to identify and synthesise the thematic components shaping the CE landscape across the UK.
B. **Review and Survey Existing Regional, City, and Sectoral CE Roadmaps and Strategies**
  Conduct an extensive review of existing CE roadmaps and strategies at national, regional, city, and sectoral levels. This objective includes collecting and analysing data on past and present efforts as well as projecting future trajectories. The survey will also involve identifying key CE practices (the “Rs”) and actors, including leading sectors, stakeholders, priority areas, action plans, metrics, and existing KPIs, providing a comprehensive overview of ongoing CE efforts in the UK across different scales.
C. **Propose Strategic Parameters for a National CE Roadmap**
  Use the findings from objectives A and B to define a set of strategic parameters that will guide the development of a national CE roadmap for the UK, outlining the roles of different stakeholders, suggest mechanisms for collaboration across sectors and regions, and propose governance structures that ensure effective implementation and monitoring of the roadmap.

## Literature Review

The UK’s strategic shift towards a CE is driven by the imperative to mitigate the impacts of resource depletion and waste generation while fostering innovation and competitiveness in a rapidly evolving global market [2]. The literature on CE practices across all regions in the UK highlights several critical themes, including regulatory frameworks and governance, sectoral and value chain strategies, technological innovations, barriers to implementation, stakeholder engagement, and metrics and key performance indicators (KPIs). These themes collectively underscore the strategic imperatives and practical steps necessary for developing a cohesive national CE roadmap, reflecting the unique challenges and opportunities across the UK’s diverse regions and sectors.

### Regional Strategic Interventions

Regions across the UK focus on systematic transitioning into a circular model through hierarchical strategic interventions [30]. Taking the automotive sector as an example, the refusal strategy eliminates unnecessary products at the design stage, reducing waste, as seen in cutting plastic packaging in automotive parts [31]. Reduction enhances material and energy efficiency, exemplified by lightweight materials in vehicle manufacturing [32]. Reuse involves direct re-utilisation, such as certified pre-owned car programs [33]. Repair extends product life by fixing faults, like repairing headlights [34]. Refurbishment improves and resells older products, such as updating car models or engines [35]. Remanufacturing disassembles and reassembles products to original specs, shown in remanufacturing transmissions [36]. Recycling processes materials into new products, exemplified by recycling metals from scrapped cars [31]. Recovery extracts energy or materials from waste, such as incinerating car shredder residue for energy [32].

### Governance and Policy Frameworks

Regulatory frameworks and governance structures are pivotal in implementing these practices, exemplifying a diverse and regionally tailored approach to CE implementation [15]. The Waste Framework Directive and the Landfill Directive have significantly influenced the transition from a linear to a circular waste management model [37]. The principle of refusing unnecessary products aligns with UK policy efforts to minimise waste generation at the source by eliminating non-essential items in design processes. For instance, reducing the use of plastic packaging for car parts exemplifies this approach. The UK government’s Plastic Packaging Tax, introduced in April 2022 and set at £200 per tonne, incentivises businesses to use recycled plastic in packaging. This policy is part of a broader strategy to eliminate avoidable plastic waste by 2042 [38, 39]. The UK Plastics Pact, also targets the elimination of problematic or unnecessary single-use packaging through redesign, innovation, or reuse models, further supporting the reduction of plastic use in various sectors, including automotive parts [39]. Benchmarking against international leaders highlights gaps in current UK frameworks. For instance, the Netherlands’ 2016 Transition Agenda mandates that each sector halve its consumption of primary raw materials by 2030 [40] and Finland’s National Public Procurement Strategy requires that at least 25% of government spendin adhere to circular-economy criteria, thereby stimulating demand for circular products and services [41]. Such ambitious upstream targets are absent from existing UK policy, suggesting an opportunity to adopt more aggressive measures that drive virgin-material avoidance and circular procurement.

### Collaborative Models

The Resource Recovery from Waste (RRfW) programme illustrates successful collaboration among academia, government, and industry, co-creating a vision for sustainable waste and resource management [42].

Scotland’s policy framework integrates product design for durability, repair, upgrade, and remanufacture into national strategies, emphasising the complete disassembly and reassembly with new parts to meet original specifications [43]. Using the ‘Double Diamond’ framework, the process identified 114 actors and initiatives related to design and CE, crucial for aligning market and government needs. Scotland’s Zero Waste Plan targets recycling 70% of all waste by 2025 and reducing landfill waste to 5% by 2025, showcasing ambitious environmental goals [16], comprehensively underscoring the value of repair and remanufacture strategies in extending product lifecycles.

### National and Regional Policy Alignment

Policy support at various scales, including the European CE Action Plan and the UK’s own CE Package, is crucial for aligning economic and environmental goals, such as carbon neutrality and sustainable growth [24, 30, 44]. National policies like the UK Industrial Strategy, the Resource and Waste Strategy, and the Decarbonisation Strategy emphasise the importance of the CE for reducing carbon emissions and achieving a more sustainable society [25]. However, regional policies must balance local economic benefits with national directives, necessitating strong local-national coordination to effectively implement CE initiatives [30]. In Wales, the Towards Zero Waste strategy aims for zero waste by 2050, with an interim target of 70% recycling by 2025 [17]. Wales’ approach is highlighted by 21 case studies across various sectors, such as food surplus redistribution by Aber Food Surplus and advanced steel recycling by Celsa Steel UK, underscoring the importance of community engagement and regional collaboration in achieving CE goals [45]. Newsholme et al. [30] also highlight the emphasis on recycling as a prominent end-of-life strategy in the CE, with specific initiatives such as Arco’s focus on packaging waste management in North Humberside and national policies promoting the economic viability of secondary raw materials. The focus on recycling and repurposing materials illustrates the practical application of these hierarchical practices within local contexts. Using old tyres for playground surfaces demonstrates innovative repurpose of products for different functions [31, 46]. Legal mandates require businesses to manage waste responsibly; however, 25% of SMEs were found to be illegally using household waste services, indicating significant non-compliance and resource leakage [47]. Addressing these issues is crucial for enhancing recycling rates, particularly as the UK produces approximately 2 million tonnes of e-waste annually [48]. The emphasis on reducing and reusing products within the regulatory framework aligns with broader CE objectives of efficiency in material and energy use. The automotive industry’s adoption of lightweight materials to reduce vehicle weight epitomises efforts to minimise resource consumption during manufacturing [32, 33].

Scotland’s CE plan, developed through collaboration with 114 stakeholders, led to 12 actionable policies that effectively address resource scarcity and environmental challenges, demonstrating the power of diverse input in creating practical, impactful solutions [43]. Similarly, regional collaboration in the Southwest UK involving business, governance, and community groups drives CE adoption by leveraging local knowledge and resources [49]. Local authorities, industry leaders, and community groups are pivotal in driving CE initiatives, as demonstrated by collaborative projects that integrate CE principles into diverse sectors, highlighting the necessity of regional partnerships. The success of these initiatives, particularly in areas like e-waste management and community-supported agriculture, underscores the critical role of engaging multiple stakeholders across public, private, and third sectors to achieve substantial environmental and socio-economic benefits through effective collaboration [45, 48].

Despite the successes of stakeholder collaboration in advancing CE initiatives, critical gaps remain, particularly in aligning regional and corporate objectives to ensure cohesive implementation. Newsholme et al. [30] identify a significant misalignment between regional policymakers and businesses, with regional authorities emphasising local economic and environmental benefits while seeking national support, whereas businesses focus on internal operations and global supply chains with little regional engagement. This disconnect necessitates more integrated approaches that consider both regional and corporate perspectives. Effective governance frameworks should incentivise local collaborations and align corporate practices with regional objectives to overcome these barriers. Thus, while stakeholder engagement is crucial, overcoming the misalignment between policy and practice is essential for the successful implementation of CE initiatives.

### Sector-Specific Practices

Sectoral and value chain strategies emphasise tailoring CE practices to the specific requirements of different industries. The manufacturing sector exemplifies leadership in integrating CE principles through remanufacturing and refurbishment, leveraging Industry 4.0 technologies such as IoT and AI to enable real-time monitoring and predictive maintenance [50]. The National Industrial Symbiosis Programme (NISP) serves as a model for facilitating material exchanges between companies, enhancing resource efficiency and reducing waste. In the retail sector, companies like M&S and Tesco drive CE initiatives focused on waste reduction, sustainable sourcing, and recyclable packaging, responding to consumer demand for sustainability and regulatory compliance [51]. M&S, for example, has set ambitious targets to achieve zero waste disposal by 2025 and ensure 100% recyclable packaging by 2030. The food supply chain demonstrates CE integration through the reuse of agricultural by-products and assurance schemes promoting CE adoption. ReFood anaerobic digestion plants, which convert food waste into energy and fertiliser, exemplify effective resource recovery practices [52]. In Wales, Celsa Steel UK’s annual recycling of 1.2 million tonnes of scrap metal into new steel products underscores the potential for creating closed-loop systems and strategic partnerships to enhance circularity [45]. These sector-specific strategies leverage technological advancements and collaborative efforts across value chains to address unique challenges and opportunities. The reuse strategy is illustrated by initiatives like certified pre-owned programs in the automotive industry, facilitating the direct re-utilisation of products without reprocessing.

### Technological Innovations

Technological innovations are pivotal to advancing CE by enhancing efficiency and sustainability across industries. Industry 4.0 technologies, including big data, artificial intelligence, and IoT, revolutionise manufacturing processes, enabling predictive maintenance and optimising remanufacturing and refurbishment [50, 53, 54]. These technologies improve resource management and operational efficiency through real-time data and automated quality checks. For instance, lean manufacturing practices in the automotive sector demonstrate how technological advancements can reduce material and energy consumption [32]. The integration of advanced multi-criteria decision-making tools, specifically the stepwise weighted influence nonlinear gauge system (WINGS) method and Fermatean fuzzy linguistic sets, by London Metropolitan optimises e-waste management by comprehensively evaluating social, technical, environmental, and policy-related factors [48]. Material Flow Analysis (MFA) models, which study rare earth elements (REEs), provide critical insights into REE flows and stocks, essential for developing effective recycling and refining capacities [23]. Scotland’s significant investment in smart waste management systems has notably improved resource recovery rates. The development of a value chain-based taxonomy for CE KPIs underscores the need for a systematic approach to measuring and managing CE performance [55]. Technological innovations are thus essential in overcoming traditional barriers to CE implementation and fostering innovation across supply chains, enabling the identification of critical gaps and opportunities for enhancing CE infrastructure.

Emergent enablers, such as Digital Product Passports (DPPs) mandated under the EU’s Eco-design for Sustainable Products Regulation (EU 2023/424) and currently piloted in the UK electronics and textiles sectors during 2024–25, operate alongside the nine-R hierarchy by providing granular traceability data that can unlock new reuse, repair and high-value recycling pathways, especially for complex products [56, 57].

### Barriers To Implementation

Barriers to implementation of the CE in the UK encompass economic, technological, and socio-political challenges. High initial costs and uncertain returns on investment deter businesses from adopting CE practices, while the need for advanced infrastructure and expertise poses significant technological barriers, particularly for small and medium-sized enterprises [58]. In England, 25% of SMEs were found to be illegally using household services for waste disposal, highlighting compliance issues and resource leakage [47]. Organisational resistance to change and the complexity of aligning new circular practices with existing regulations represent socio-political barriers [51]. Data availability and traceability issues hinder effective material flow management, as demonstrated in the study of rare earth elements, where inconsistent data across the supply chain limits robust CE strategy development [23]. The study found that 6% of household waste sampled originated from SMEs, with 77% of this waste being biowaste or dry recyclable materials that could have been diverted through recycling programs [47]. Regional disparities in policy implementation and support create uneven progress across the UK, highlighting the need for coherent national strategies that provide financial and technical assistance to local authorities [59]. Overcoming these barriers requires targeted financial incentives, capacity-building initiatives, and enhanced stakeholder collaboration. Addressing legal and regulatory frameworks is crucial for supporting CE practices, while innovation and investment in new technologies and business models are essential for overcoming these barriers.

### Metrics and KPIs

Robust metrics and KPIs are critical for evaluating the progress and effectiveness of CE practices. Effective metrics provide insights into resource efficiency, waste reduction, and the environmental impact of CE initiatives. The development of a value chain-based taxonomy for CE KPIs emphasises the necessity of a systematic approach to performance measurement, integrating horizontal value chains, vertical scales of operation, and various impact categories [23, 29, 55]. Standardised and widely accepted KPIs facilitate cross-sectoral learning, enhance transparency, and drive continuous improvement in CE practices.

### Summary of Key Findings

The literature review provides crucial insights into how regional strategies, governance, collaboration, technology, and sector-specific approaches influence the shift to a CE in the UK. Table 1 outlines the key themes and their relevance to developing a scalable CE framework for the UK. Most UK studies concentrate heavily on recycling rates as the primary measure of circularity, often neglecting upstream indicators such as virgin-material avoidance, product-service substitution or circular business models [30]. Moreover, there is a tendency to rely on policy analyses and roadmap descriptions without systematically testing their real-world efficacy; empirical evaluations, particularly on social-equity outcomes and long-term resource savings, remain scarce. Methodological limitations include inconsistent indicator definitions across regions, limited triangulation with primary data (e.g., stakeholder interviews or case studies) and inadequate attention to non-policy drivers such as cultural practices and market dynamics. These gaps and contradictions highlight the need for a more rigorous, replicable approach that can benchmark diverse CE practices on a uniform set of metrics.

**Table 1:** Summary of key literature findings

Theme	Key Findings	References
Regional Strategic Interventions	CE principles such as refusal, reuse, and recycling are applied in various sectors, particularly in the automotive industry.	[26–29]
Governance and Policy Frameworks	UK regulatory frameworks (e.g., Plastic Packaging Tax) promote waste reduction and incentivise recycling across sectors.	[9, 25, 33, 34]
Collaborative Models	Scotland’s Zero Waste Plan shows that collaboration between academia, industry, and government leads to better CE outcomes.	[35, 36]
National and Regional Policy Alignment	Disparities between national and regional policies highlight the need for better coordination in CE efforts across the UK.	[19, 25]
Sector-Specific Practices	Sectors like manufacturing and retail integrate CE through remanufacturing and waste reduction strategies, supported by Industry 4.0 technologies.	[38, 43–45]
Technological Innovations	Industry 4.0 technologies enhance CE by improving resource efficiency and enabling predictive maintenance.	[27, 41, 47, 48]
Barriers to Implementation	Economic, technological, and socio-political barriers, including high costs and resistance to change, impede CE adoption.	[40, 49, 50]
Metrics and KPIs	Standardised KPIs are crucial for tracking CE performance and ensuring accountability across sectors and regions.	[18, 23, 48]

## Materials and methods

### Review and Survey of Regional, City, and Sectoral CE Roadmaps

The study employed an NLP framework to analyse a selection of CE documents across multiple governance levels in the UK, including national, regional, city, and sectoral initiatives. The goal was to extract and categorise key CE principles, refuse, reduce, reuse, repair, refurbish, remanufacture, repurpose, recycle, and recover, to understand how these principles are implemented and prioritised across regions and sectors. Named Entity Recognition (NER) [60] and custom text-processing techniques were applied to identify patterns in CE practices, highlighting regional differences and sectoral strategies, as well as the roles of various stakeholders in the UK’s CE efforts; manual double-coding of a 10% plot sample (Cohen’s κ = 0.83) confirmed acceptable reliability but would have required roughly 900 analyst-hours to complete the full corpus, so spaCy-based NER pipelines were adopted to ensure the necessary consistency, speed and auditability.

### Document Collection and Preprocessing

A total of 36 documents were identified for analysis based on their relevance to long-term CE planning [28, 29], following a structured inclusion and exclusion process. These documents were identified through public repositories, government websites, and sector reports, ensuring a comprehensive representation of CE strategies across different governance levels and sectors in the UK. Text from the documents was extracted using Python’s python-docx library and segmented into paragraphs for detailed analysis. This preprocessing step enabled a fine-grained examination of CE practices and ensured that the data was prepared for subsequent NLP-driven analysis. The inclusion and exclusion criteria used to ensure the relevance and comprehensiveness of the selected documents are outlined in Table 2.

**Table 2:** Document selection criteria

Criteria	Inclusion	Exclusion
Relevance	Documents must articulate a long-term vision for CE, including strategic goals, comprehensive timeline, and actionable plans within the UK context.	Documents lacking a strategic perspective or focused solely on short-term operational issues without broader CE planning.
Governance Level	Must represent a diverse range of UK governance levels, including national, regional, city, and sector-specific initiatives, ensuring a comprehensive understanding of CE practices across different administrative contexts.	Documents not aligned with the UK governance structure or focused on non-UK CE practices, limiting their applicability to the UK context.
Comprehensive Focus	Should provide a broad and integrative view of CE strategies, encompassing multiple sectors and stakeholder perspectives to inform a wide-ranging analysis.	Documents with a narrow technical focus or that do not extend beyond specific technical aspects of CE, thereby lacking broader strategic implications.
Authorship & Provenance	Authored or officially endorsed by a recognised UK government department or statutory agency, a devolved administration, or a sectoral/city authority.	Internal memos, unpublished consultancy presentations or stakeholder meeting minutes without formal publication credentials.
Date Range	Published between January 2010 and December 2024, ensuring coverage of both early and recent CE evolutions.	Pre-2010 publications or draft documents without a clear publication date.
Accessibility	Must be publicly accessible through reputable sources such as government websites, industry reports, or academic publications, ensuring transparency and the ability for future researchers to access the data.	Documents not publicly accessible or behind restrictive paywalls, limiting the ability for peer verification and further academic scrutiny.

### NLP Techniques

#### NER

NER [60] was used to identify and categorise entities within the text, including locations, sectors, stakeholders, and CE principles. This technique is effective in converting unstructured text into structured data, essential for large-scale analysis [61–63]. A predefined lexicon was used to extract relevant data points [64]. Custom scripts were developed to detect and count mentions of the “R” principles and categorise stakeholders using regular expressions. This enabled precise identification of CE practices and their regional and sectoral contexts.

#### Data Structuring and Analysis

Extracted entities were organised into a data frame with variables such as location, CE principles, sectors, sub-sectors, and stakeholder categories. This structure supported qualitative and quantitative analysis, identifying patterns and regional disparities in CE practices.

### Rationale for Method Selection

NLP and NER were selected for their scalability and ability to process extensive and varied textual data, reducing biases associated with manual content analysis [62]. These methods have been validated in similar policy document analyses, proving effective in academic research. Previous studies, such as [65] have successfully used NLP and NER to analyse policy documents, demonstrating these techniques’ effectiveness in uncovering regional and thematic trends.

### Limitations of Policy-Document Analysis

Despite applying clear inclusion and exclusion criteria to select 36 publicly available CE roadmaps and strategies, relying exclusively on policy documents may introduce biases: these texts often emphasise intended goals rather than actual implementation, can omit recent or unpublished initiatives (e.g., corporate or local pilot programmes), and reflect the priorities of their authors. Although our structured selection process minimised arbitrary choices, this approach cannot fully eliminate the potential for policy-language bias or gaps in emerging practice.

## Results

The dataset, derived from the collective quantified insights from 36 roadmaps and strategy documents across the UK, reveals underlying patterns that reflect the strategic vision and operational mindset driving both current and future CE practices. Spanning 22,589 entries, it captures key CE activities alongside broad sectoral and stakeholder engagement, revealing the diverse involvement across industries and regions. Table 3 provides a concise overview of the distilled variables, offering contextual definitions and their relevance to the broader analysis.

**Table 3:** Distilled variables and their relevance

Variable	Definition	Relevance	Unique Instances
Location	Geographic area of CE practice (e.g., city, region, UK-wide).	Identifies regional CE distribution, enabling comparative analysis across different areas.	52
R (CE Practices)	Specific CE activities along the CE value hierarchy (e.g., refuse, reuse, recycle).	Reveals the prioritisation of CE practices in retaining value within the economy, guiding strategic focus.	9
Sector	Broad industry sectors involved in CE (e.g., Manufacturing, Construction).	Highlights the main economic sectors where CE is applied, allowing for sector-specific insights.	34
Sub-Sector	Specific categories within broader sectors (e.g., Food Waste, Sustainable Production).	Provides detailed insights into niche areas, supporting the development of targeted CE strategies.	62
Stakeholder Category 01	Primary stakeholders (e.g., businesses, government, NGOs).	Identifies key actors driving CE initiatives, crucial for designing effective stakeholder engagement strategies.	109
Stakeholder Category 02	Secondary stakeholders (e.g., academia, investors).	Shows the broader network of supporting actors, highlighting collaborative opportunities within CE initiatives.	78
Stakeholder Category 03	Tertiary stakeholders (e.g., media, consultants).	Highlights indirect influencers, underscoring the importance of engaging with the wider stakeholder landscape.	56

### Overview of Circular Practices across the UK

Across the UK, recycling remains the most prevalent circular practice, accounting for 42.8% of all activities. This is followed by reduction at 29.7%, indicating a strong commitment to minimizing waste generation. Recovery practices contribute 8.79%, while repair accounts for 8.56%. The practices of reuse, remanufacture, refurbish, and rethink collectively make up 10.16% of CE efforts, with reuse at 3.12%, remanufacture at 2.91%, refurbish at 2.5%, and rethink at 1.63%.

In England, recycling constitutes 46.5% of all circular practices, while reduction accounts for 32.5%. Recovery makes up 8.94%, and repair represents 5.4%. The remanufacture, refurbish, reuse, and rethink practices together account for 6.58% of circular efforts, with reuse at 2.19%, refurbish at 1.52%, and rethink at 0.169%.

In Scotland, recycling contributes 45.7% of circular practices, followed by reduction at 29.1%. Repair is prominent at 11.2%, showing a strong focus on extending product life. Recovery accounts for 4.59%, and remanufacture contributes 4.46%. The remaining practices, reuse, refurbish, and rethink, collectively account for 4.995%, with reuse at 2.76%, refurbish at 1.71%, and rethink at 0.525%.

In Wales, recycling dominates at 53.5% of circular activities, followed by reduction at 21.6%. Repair accounts for 10.6%, and recovery makes up 6.53%. Remanufacture, reuse, refurbish, and rethink collectively make up 7.756%, with reuse at 1.63%, refurbish at 2.04%, and rethink at 0.816%.

In Northern Ireland, recycling constitutes 44.3% of circular practices, while reduction follows at 27.8%. Repair is relatively high at 11.3%, reflecting a focus on extending product life. Recovery accounts for 5.15%, and remanufacture and reuse both make up 3.09% each. Refurbish is notably higher at 4.12%, and rethink constitutes 1.03% of the overall practices.

**Fig. 1:**
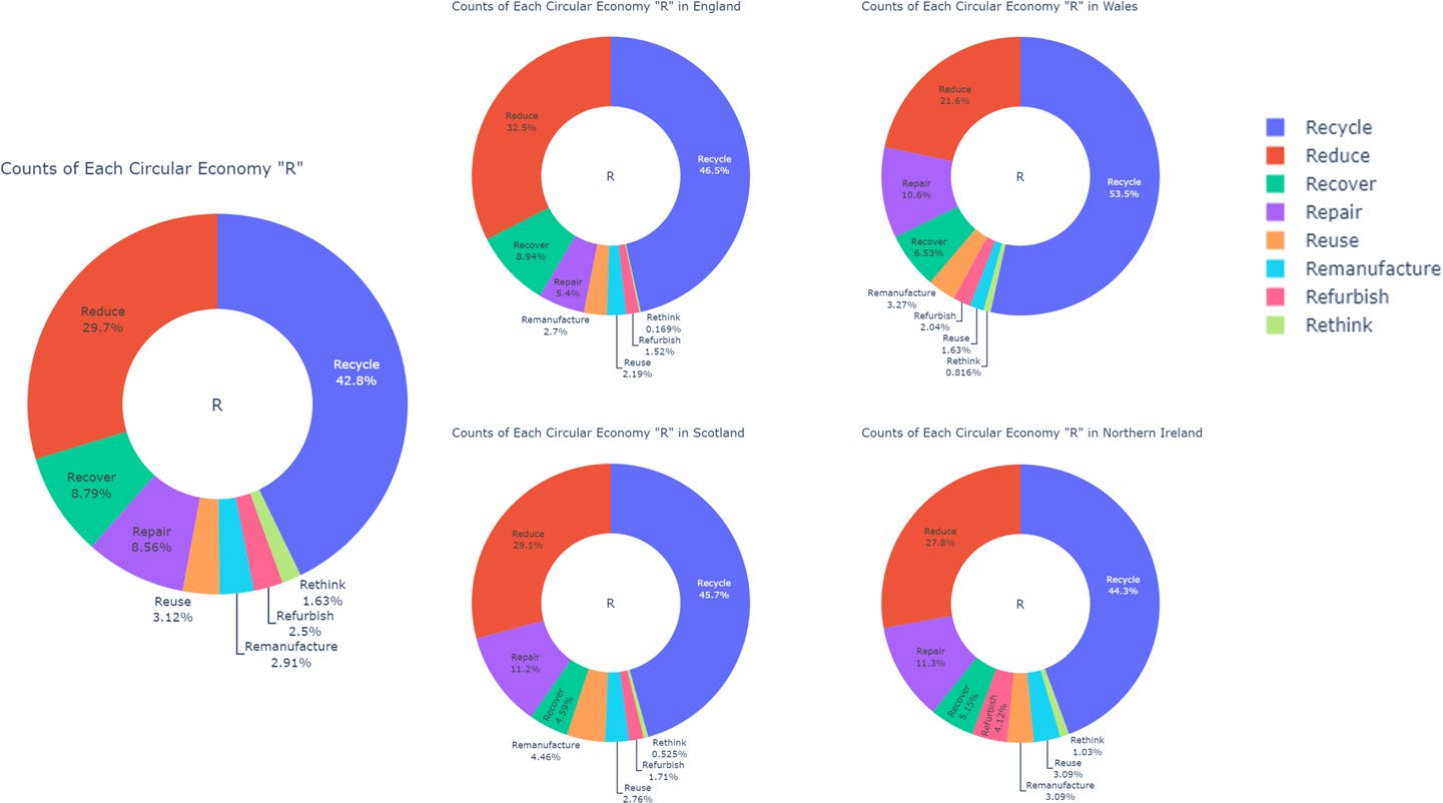
Distribution of CE Practices (“Rs”) Across the UK

Figure 1 illustrates CE practice patterns across the UK.

### Overview of Sectors across the UK

Across the 32 identified sectors, Construction & Demolition accounts for 19.8% of all CE initiatives. The Food & Beverage sector follows with 13.7% of initiatives. Manufacturing represents 10.3%, while Rare Earth Metals contributes 9.8%. The Plastics sector accounts for 8.8% of total initiatives.

Other notable sectors include Metals at 8.4%, Energy Systems at 7.8%, and Packaging at 5.5%. Additional sectors such as Transport & Logistics and Fossil Fuel represent 4.1% and 4.0%, respectively.

Sectors like Healthcare & Pharmaceuticals (2.3%), Water Utilities (1.9%), and Agriculture (2.0%) contribute smaller portions of initiatives. Sectors including Aviation, E-Waste, Furniture, and Textiles each make up less than 2% of total initiatives.

**Fig. 2:**
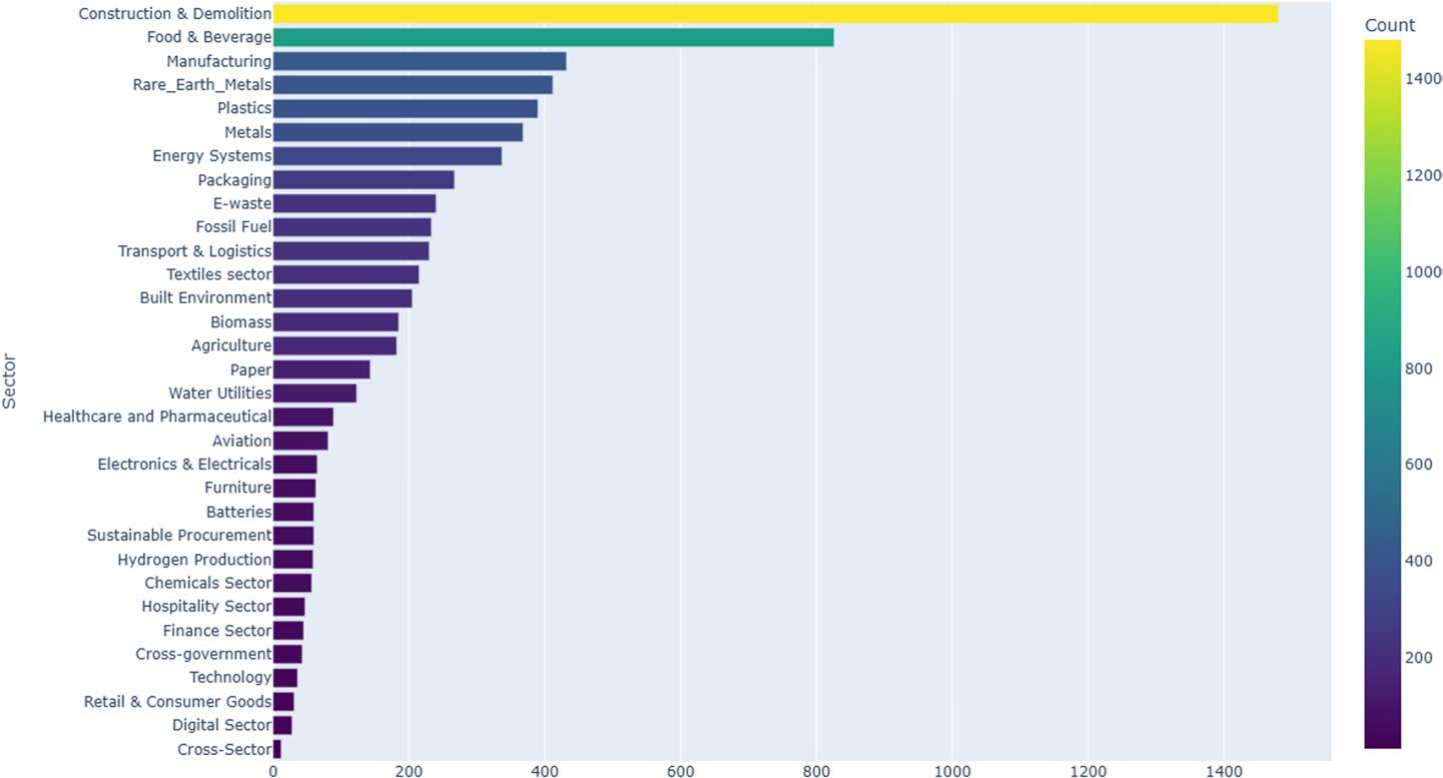
Sectors Distilled from CE Documents in the UK

Figure 2 provides a visual representation of the distribution of CE initiatives across all sectors.

As shown in Table 4, sectoral distribution across the four nations presents distinct focuses. Construction & Demolition accounts for 16.58% of initiatives in England, 6.08% in Scotland, 10.53% in Wales, and 11.01% in Northern Ireland. The Food & Beverage sector contributes 4.94% of initiatives in England, 8.51% in Scotland, 10.12% in Wales, and 6.47% in Northern Ireland.

**Table 4:** Sectoral distribution

Identified Sectors	England (%)	Scotland (%)	Wales (%)	Northern Ireland (%)
Construction & Demolition	16.58	6.08	10.53	11.01
Energy Systems	6.19	1.07	0.41	3.89
Food & Beverage	4.94	8.51	10.12	6.47
Transport & Logistics	4.27	0.63	0.41	0
Water Utilities	3.61	0.09	0	1.29
Manufacturing	3.44	3.54	3.25	4.56
Plastics	3.44	1.51	1.62	0.66
Textiles sector	2.59	1.68	0	1.96
Rare_Earth_Metals	2.36	2.31	3.25	3.92
Fossil Fuel	1.93	0.09	0.82	1.29
Built Environment	1.69	2.39	8.12	0
Furniture	1.6	0.53	0	2.58
Metals	1.52	1.24	6.09	2.58
Biomass	1.1	1.33	0.41	0
Agriculture	1.03	0.71	1.62	7.71
Aviation	0.77	0	0	0
E-waste	0.77	0	0.41	0
Electronics & Electricals	0.68	0.18	0.82	2.58
Healthcare and Pharmaceutical	0.68	0.46	2.85	1.93
Chemicals Sector	0.59	0.65	0	0
Finance Sector	0.52	0	0	0
Hospitality Sector	0.43	0.28	2.44	0
Sustainable Procurement	0.36	0.65	0	0.66
Batteries	0.26	0	0	0
Cross-Sector	0.17	0.09	0	0
Paper	0.17	0.83	0	2.58
Cross-government	0.09	0	0	0.66
Digital Sector	0.09	0.09	0	0.66
Hydrogen Production	0.09	0.28	0.41	0
Packaging	0.09	1.07	3.68	0
Technology	0.09	0.28	0.82	0
Retail & Consumer Goods	0	0.46	1.62	0

Energy Systems represent 6.19% of initiatives in England, 1.07% in Scotland, 0.41% in Wales, and 3.89% in Northern Ireland. Meanwhile, Transport & Logistics makes up 4.27% in England, 0.63% in Scotland, 0.41% in Wales, and 0% in Northern Ireland.

Manufacturing initiatives range from 3.44% in England, 3.54% in Scotland, 3.25% in Wales, to 4.56% in Northern Ireland. Plastics account for 3.44% in England, 1.51% in Scotland, 1.62% in Wales, and 0.66% in Northern Ireland. The Textiles sector contributes 2.59% in England, 1.68% in Scotland, and 1.96% in Northern Ireland, while having no presence in Wales.

In terms of other sectors, Rare Earth Metals range from 2.36% in England, 2.31% in Scotland, 3.25% in Wales, to 3.92% in Northern Ireland. Fossil Fuels contribute 1.93% in England, 0.09% in Scotland, 0.82% in Wales, and 1.29% in Northern Ireland. Metals initiatives account for 1.52% in England, 1.24% in Scotland, 6.09% in Wales, and 2.58% in Northern Ireland.

Smaller sectors include Agriculture, with 1.03% in England, 0.71% in Scotland, 1.62% in Wales, and 7.71% in Northern Ireland. Healthcare & Pharmaceuticals range from 0.68% in England, 0.46% in Scotland, 2.85% in Wales, to 1.93% in Northern Ireland. Sustainable Procurement contributes 0.36% in England, 0.65% in Scotland, and 0.66% in Northern Ireland.

### Stakeholder Engagement across the UK

#### UK-Wide

**Fig. 3:**
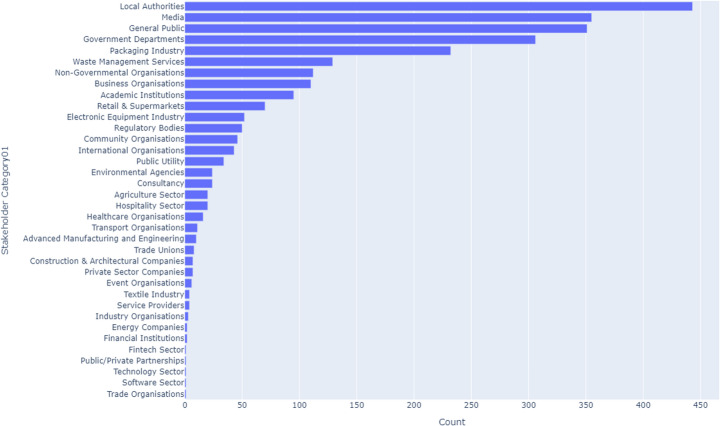
UK-wide CE stakeholders

Across the UK, Local Authorities emerge as the most engaged stakeholders in CE initiatives, accounting for 18% of activities. Following closely are the Media at 16% and the General Public at 14%. Together, these three stakeholder categories constitute nearly half of all CE activities nationwide, underscoring their significant influence on CE strategies. The Private Sector and NGOs also play critical roles, contributing 12% and 10%, respectively. In contrast, the Software Sector and Trade Organisations remain marginally involved, each representing less than 1% of activities, highlighting potential areas for increased engagement, as illustrated in Fig. 3.

#### England

**Fig. 4:**
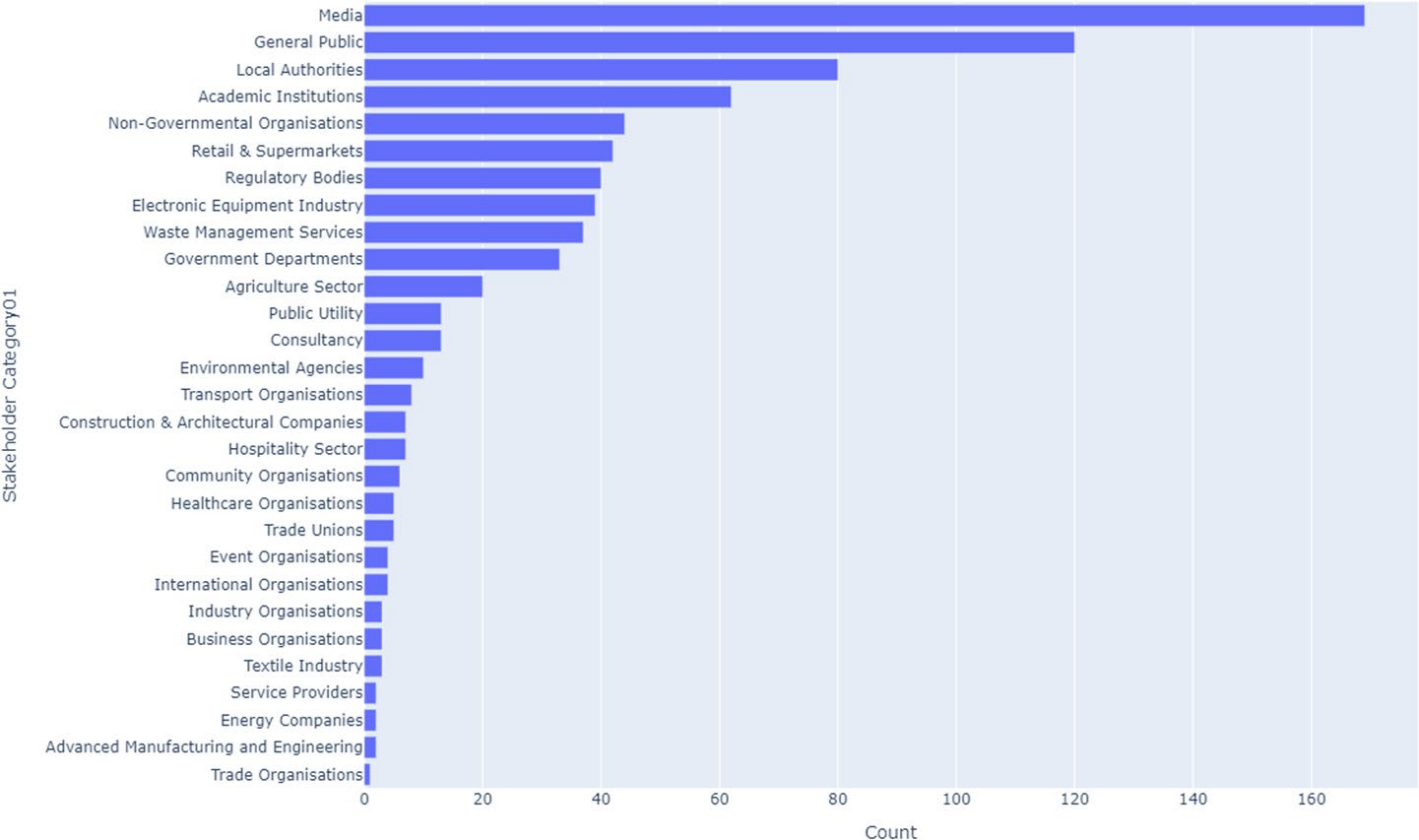
England CE stakeholders

In England, the Media leads with 11.3% of stakeholder activities, closely followed by the General Public (10.7%) and Local Authorities (10.5%). These top three stakeholders collectively represent a significant portion of CE engagement in England. Academic Institutions also play a notable role at 8.1%. However, Trade Organisations and Advanced Manufacturing and Engineering are at the bottom, each contributing less than 0.8%. The pattern in England reveals a strong media influence and public involvement, with less engagement from industry sectors that might need further integration into CE strategies. This is illustrated in Fig. 4.

#### Scotland

**Fig. 5:**
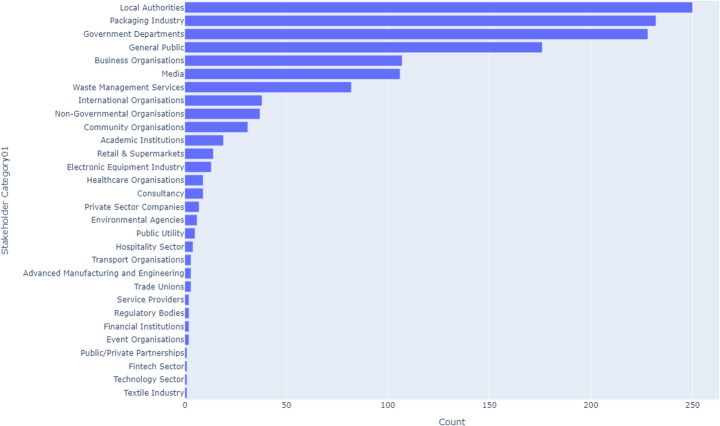
Scotland CE stakeholders

Scotland’s CE landscape is heavily influenced by Local Authorities, which account for 15.3% of stakeholder activities, the highest among all UK nations, as illustrated in Fig. 5. The Packaging Industry and Government Departments also show strong involvement, each with around 10% of activities. However, the Textile Industry and Technology Sector are minimally represented, with each contributing less than 0.5%. This pattern indicates a strong public sector presence in Scotland’s CE efforts, with specific industries like packaging playing a pivotal role, while others like textiles and technology lag behind.

#### Wales

**Fig. 6:**
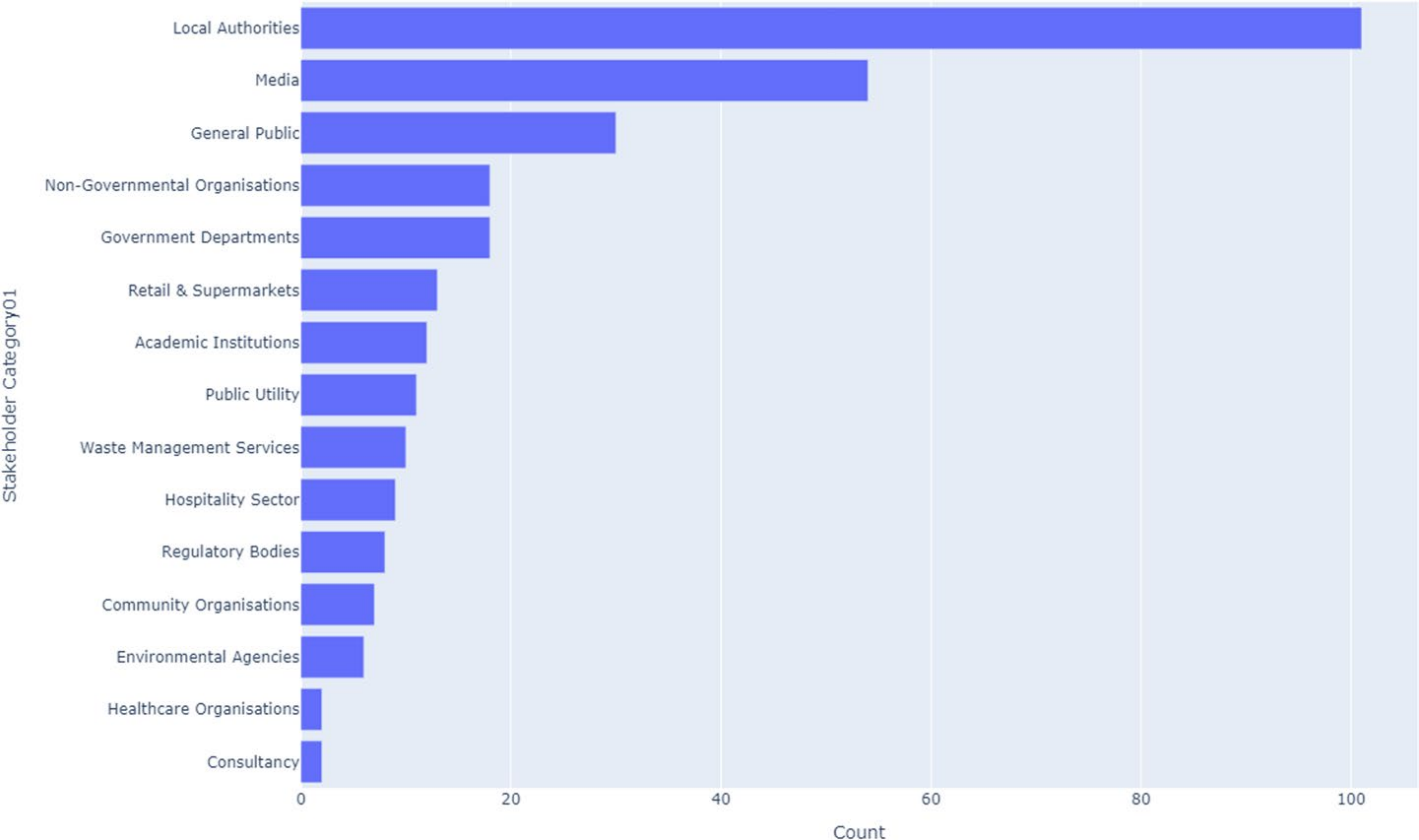
Wales CE stakeholders

Wales stands out with Local Authorities comprising 17.4% of stakeholder activities, the highest proportion in the UK, as shown in Fig. 6. Media and the General Public follow with 11.6% and 10.8%, respectively. These figures point to a well-coordinated effort between local governance and citizens. However, sectors like Consultancy and Environmental Agencies are less active, each contributing under 1.5%. The pattern in Wales highlights strong local governance and public participation but suggests that there may be opportunities to better engage specialist sectors.

#### Northern Ireland

**Fig. 7:**
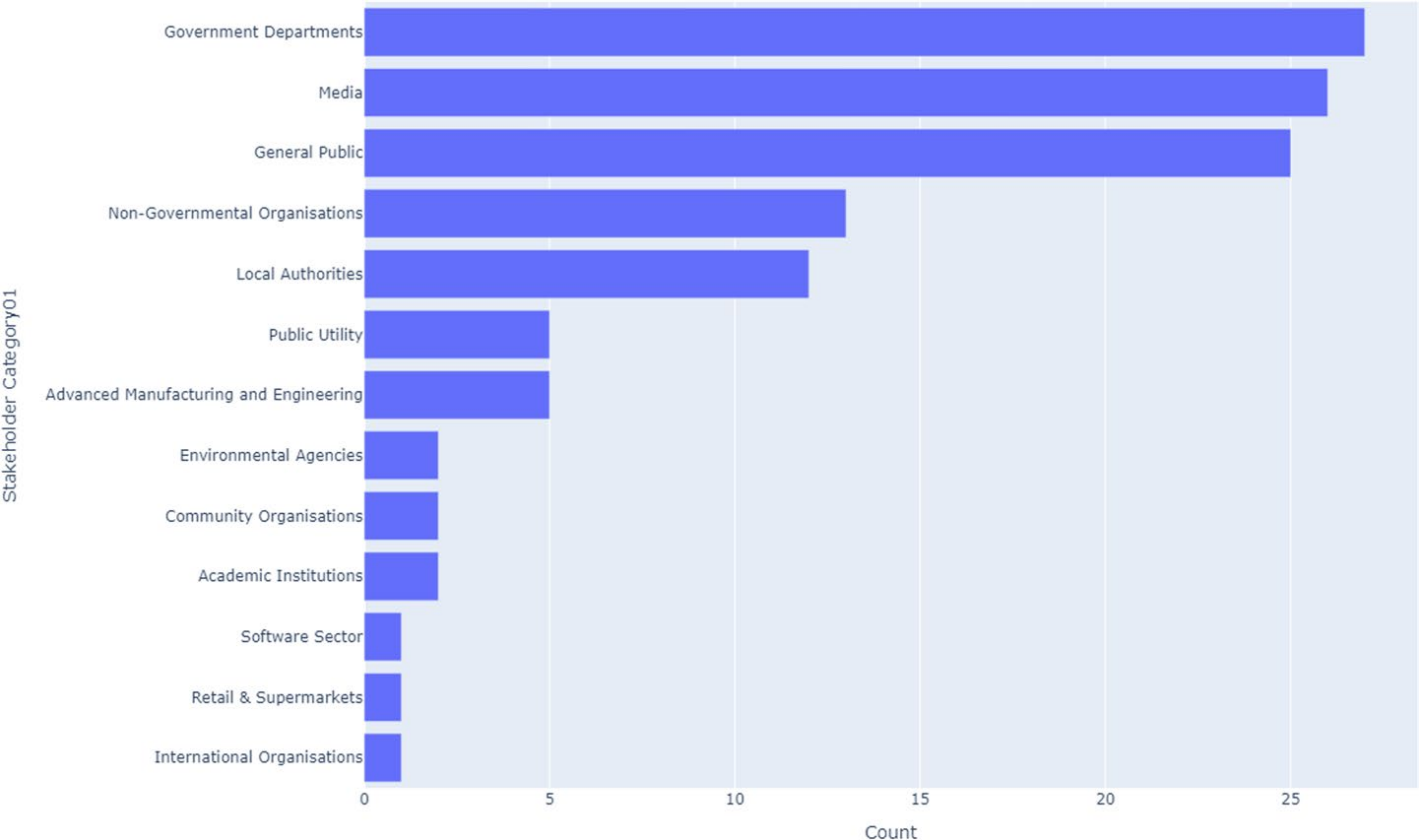
Northern Ireland CE stakeholders

Government Departments dominate CE stakeholder activities in Northern Ireland, making up 15.8% of the total, followed by Media (14.9%) and the General Public (12.3%). Advanced Manufacturing and Engineering is more active here than in other regions, contributing 6.4%. However, Retail & Supermarkets and International Organisations show minimal engagement, each at just over 0.8%. This suggests a governmental and public-centric approach to CE in Northern Ireland, with noticeable gaps in retail and international collaboration. Figure 7 Illustrates Northern Ireland’s dominant CE stakeholder.

Table 5 presents the top 10 stakeholders for across the UK, providing an insight into where CE efforts are concentrated and where further engagement might be necessary.

**Table 5:** Key CE stakeholders in the UK

Stakeholder	UK (%)	England (%)	Scotland (%)	Wales (%)	Northern Ireland (%)
Local Authorities	18	10.5	15.3	17.4	10.5
Media	16	11.3	10.0	11.6	14.9
General Public	14	10.7	8.5	10.8	12.3
Government Departments	12	8.0	10.0	9.5	15.8
NGOs	10	6.9	8.2	7.3	9.5
Private Sector	12	7.8	6.8	8.2	7.4
Packaging Industry	8	5.3	10.0	6.8	5.6
Academic Institutions	6	8.1	7.0	5.9	6.4
Advanced Manufacturing & Engineering	4	< 1	< 1	< 1	6.4
Retail & Supermarkets	2	1.2	1.5	2.0	< 1

### The Interplay between CE Practices and Sectors Across the UK

#### UK-Wide Overview

**Fig. 8:**
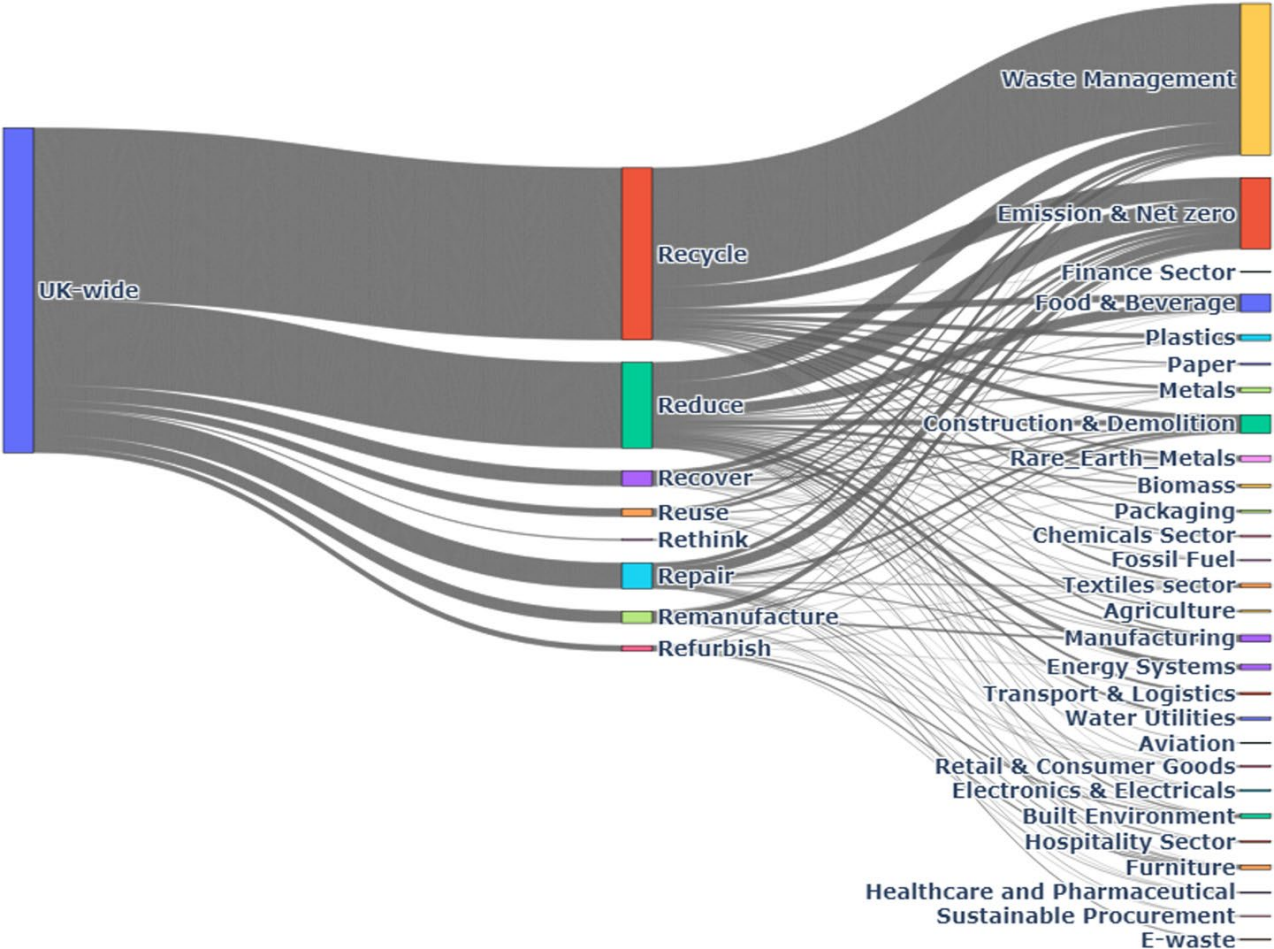
UK-wide CE practices by sector

The analysis of CE practices reveals distinct patterns of engagement between industrial sectors and the eight core Rs: Recycle, Reduce, Reuse, Recover, Remanufacture, Repair, Rethink, and Refurbish, as illustrated in Fig. 8.

Waste Management contributes significantly to recycling, with 45% of its initiatives focused on this practice. Similarly, the Manufacturing sector dedicates 30% of its CE efforts to recycling, indicating a strong focus on material recovery.

In terms of reduction efforts, the Food & Beverage sector leads, with 40% of its CE initiatives directed towards reducing resource consumption and waste generation. Construction & Demolition also shows a substantial commitment, with 25% of its activities allocated to reduction practices.

The Retail & Consumer Goods sector places a strong emphasis on reuse, dedicating 35% of its CE practices to promoting product reuse. The Textile Industry follows closely behind, with 30% of its CE efforts focused on reuse, highlighting its commitment to extending product lifecycles.

Other practices such as Repair, Remanufacture, Refurbish, Recover, and Rethink have lower engagement across sectors. Electronics and Automotive sectors show notable involvement in repair and remanufacture, with 20% and 15% of their respective CE activities focused on these practices.

#### England

In England, the Waste Management sector dedicates 50% of its initiatives to recycling, while the Manufacturing sector commits 32% of its CE efforts to the same practice.

The Food & Beverage sector leads in reduction efforts, with 42% of its initiatives focused on minimising waste and resource use.

Reuse is a significant focus for the Retail & Consumer Goods sector, which allocates 37% of its CE activities to reuse initiatives. The Textile Industry contributes 28% of its efforts to reuse, reflecting a commitment to sustainability within fashion.

**Fig. 9:**
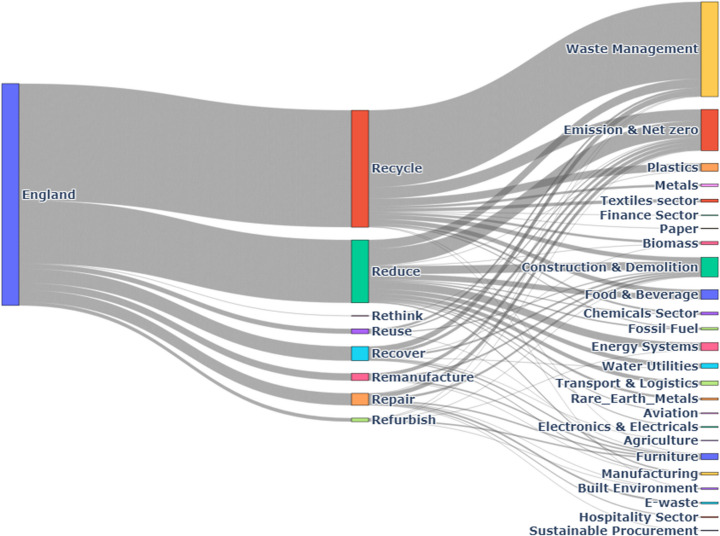
England CE practices by Sector

Repair and Remanufacture play a smaller role, with the Electronics sector dedicating 22% of its CE activities to repair. Figure 9 illustrates the relationship between CE practices and identified sectors in England.

#### Scotland

**Fig. 10:**
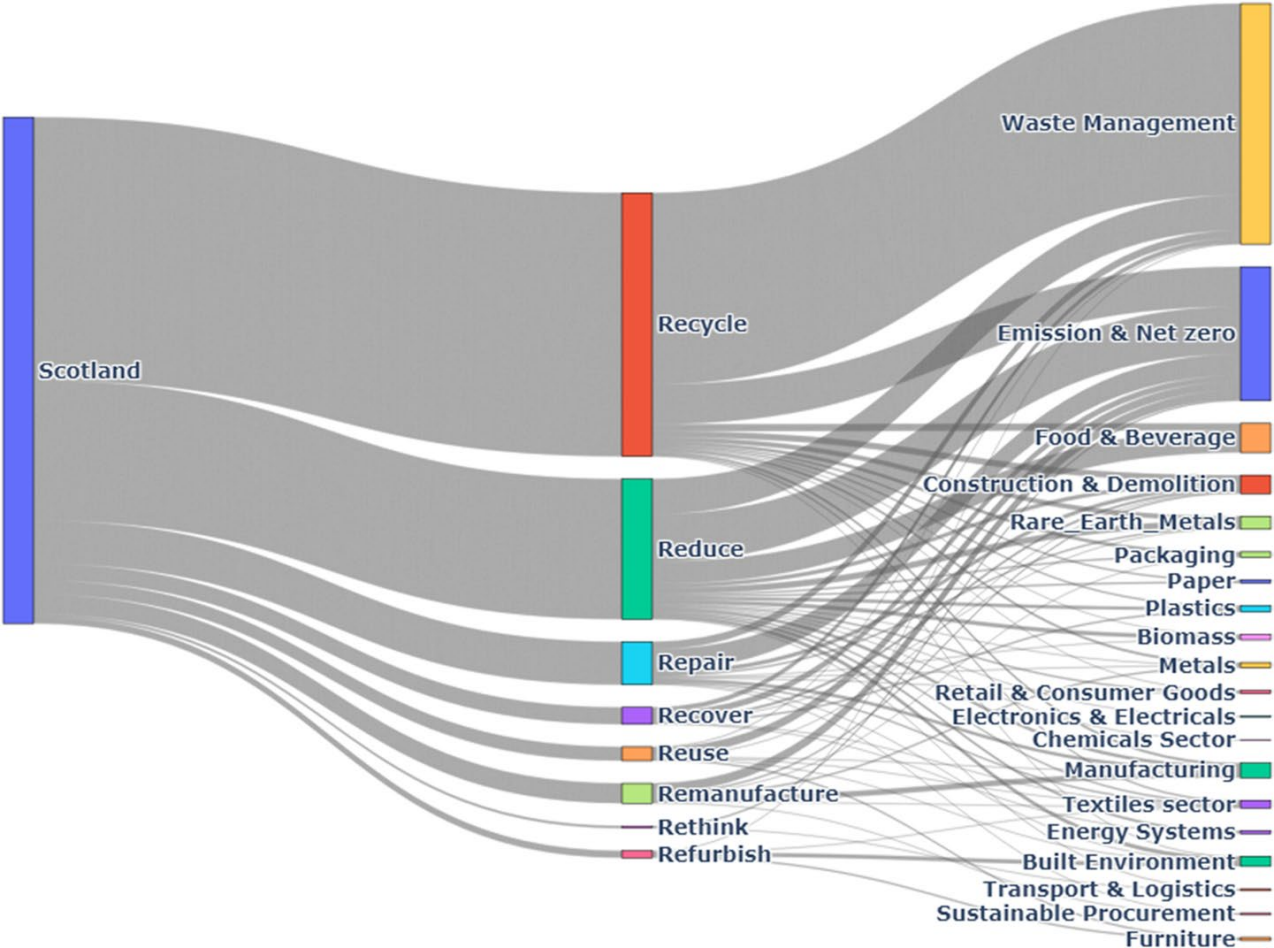
Scotland CE practices by Sector

As illustrated in Fig. 10, the Waste Management sector in Scotland dedicates 42% of its initiatives to recycling, while the Construction & Demolition sector contributes 29% to recycling efforts.

The Food & Beverage sector leads in reduction, allocating 45% of its CE initiatives to resource efficiency.

Reuse is a key focus for the Retail & Consumer Goods sector, which commits 34% of its CE activities to reuse. The Packaging Industry also shows significant engagement, with 25% of its efforts directed towards reuse strategies.

Repair and Remanufacture practices are less prominent, with the Automotive sector dedicating 18% of its initiatives to repair and 12% to remanufacture.

#### Wales

**Fig. 11:**
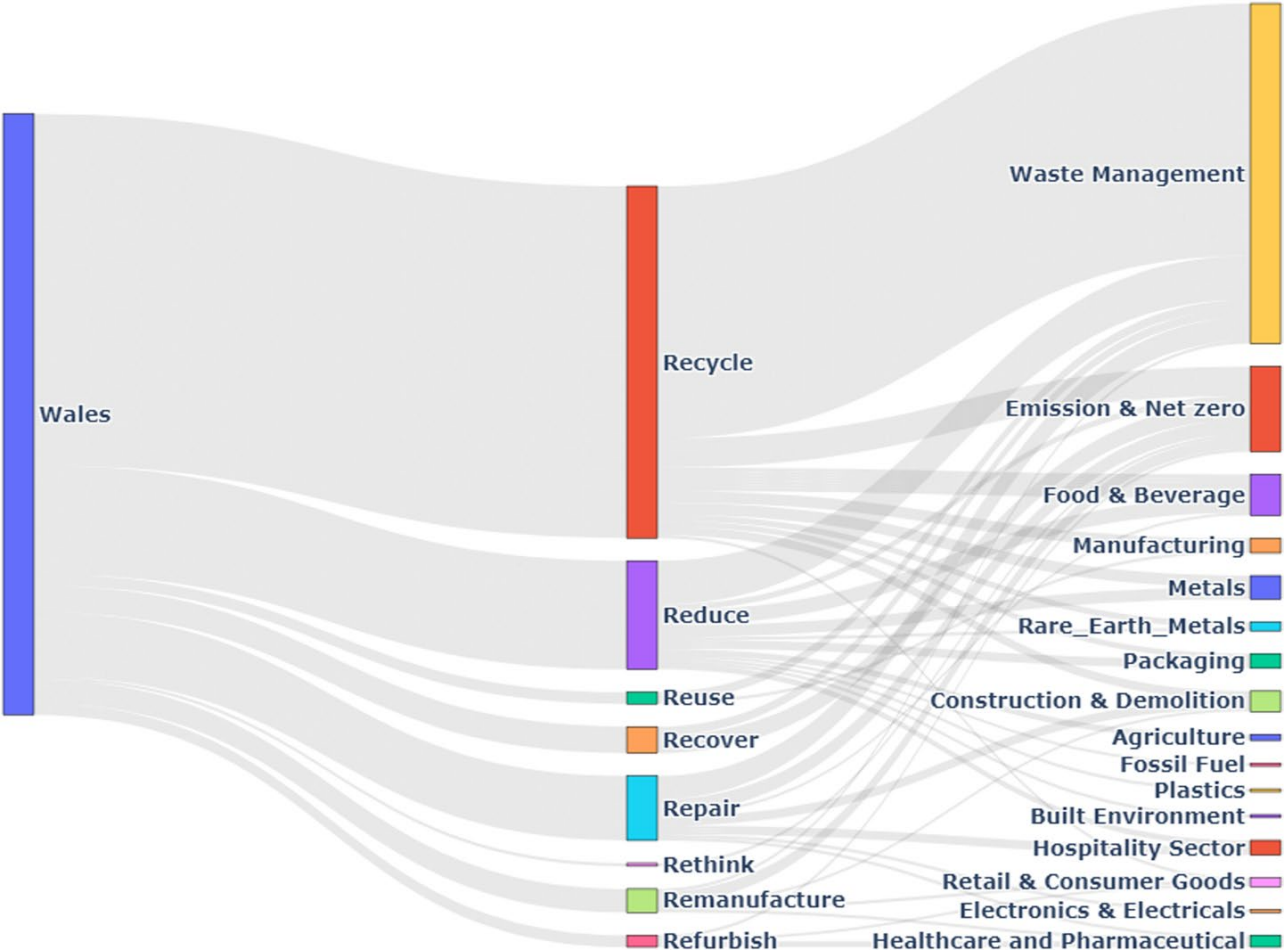
Wales CE practices by Sector

As illustrated in Fig. 11, in Wales, the Waste Management sector leads with 55% of its initiatives focused on recycling, the highest proportion among UK nations.

The Food & Beverage sector dedicates 48% of its CE efforts to reducing waste, reflecting a strong commitment to resource efficiency.

The Textile Industry plays a significant role in reuse, allocating 30% of its initiatives to reuse practices, while the Retail & Consumer Goods sector contributes 25% to reuse initiatives.

Repair and Remanufacture practices are less prominent, with the Electronics sector showing notable engagement by dedicating 20% of its CE activities to repair.

#### Northern Ireland

**Fig. 12:**
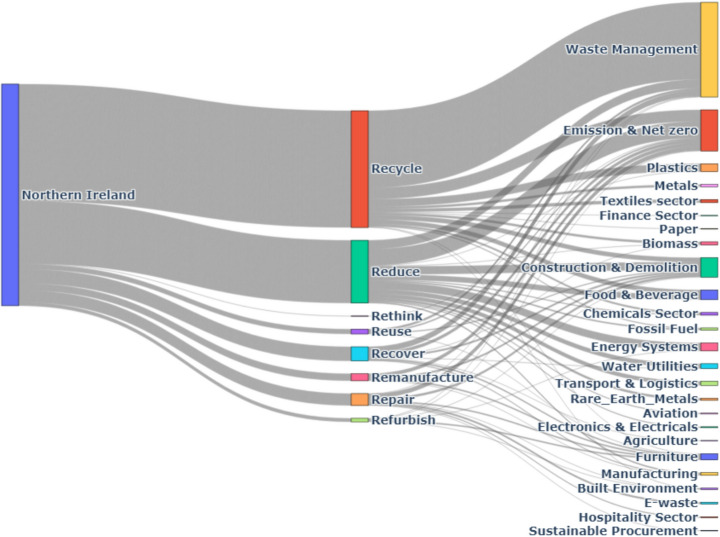
Northern Ireland CE practices by Sector

In Northern Ireland, the Waste Management sector allocates 48% of its initiatives to recycling, while the Manufacturing sector contributes 28% to recycling efforts, as shown in Fig. 12.

The Food & Beverage sector leads in reduction, with 43% of its initiatives focused on reducing resource use and waste, indicating a strong emphasis on efficiency.

The Retail & Consumer Goods sector commits 32% of its CE activities to reuse practices, while the Packaging Industry also shows significant engagement, allocating 27% to reuse initiatives.

Repair and Remanufacture play smaller roles, with the Automotive sector dedicating 19% of its activities to repair and 13% to remanufacture, highlighting efforts toward sustainable mobility solutions.

Table 6 highlights the distribution of key practices across the top five sectors in the UK and its four nations, revealing sector-specific focuses and regional priorities within the CE framework.

**Table 6:** Distribution of CE practices across top sectors in the UK and home nations

*R* (CE Practice)	Top Sectors	UK-Wide (%)	England (%)	Scotland (%)	Wales (%)	Northern Ireland (%)
Recycle	Waste Management	45%	50%	42%	55%	48%
	Manufacturing	30%	32%	29%	30%	28%
	Construction & Demolition	28%	29%	28%	25%	27%
	Energy Systems	25%	27%	25%	24%	26%
	Food & Beverage	20%	20%	20%	22%	18%
Reduce	Food & Beverage	40%	42%	45%	48%	43%
	Construction & Demolition	25%	25%	23%	24%	21%
	Manufacturing	20%	22%	21%	20%	20%
	Energy Systems	18%	19%	20%	18%	17%
	Packaging Industry	15%	16%	17%	15%	14%
Reuse	Retail & Consumer Goods	35%	37%	34%	30%	32%
	Textile Industry	30%	28%	25%	25%	27%
	Packaging Industry	25%	26%	27%	27%	27%
	Agriculture	22%	23%	24%	23%	22%
	Food & Beverage	20%	21%	19%	18%	19%
Repair	Electronics & Electricals	20%	22%	18%	20%	19%
	Automotive	15%	16%	12%	15%	13%
	Manufacturing	12%	13%	10%	12%	11%
	Aviation	10%	11%	9%	11%	10%
	Construction & Demolition	8%	9%	7%	8%	8%
Remanufacture	Automotive	15%	16%	12%	14%	13%
	Manufacturing	12%	13%	11%	12%	11%
	Aerospace	10%	11%	9%	10%	10%
	Electronics & Electricals	9%	10%	8%	9%	9%
	Packaging Industry	7%	8%	6%	7%	7%

## Discussion

The comprehensive analysis of CE practices across the UK paints a complex picture of how sectoral focus, regional disparities, and stakeholder engagement intersect to shape the nation’s journey toward a CE. These findings, when contextualized within existing literature and augmented by specific regional strategies and roadmaps, offer a nuanced understanding of the UK’s CE landscape. This discussion synthesizes the findings with technical insights from various strategies, making a strong case for a unified UK-wide CE roadmap that addresses both regional strengths and sectoral gaps.

### Sectoral Dominance and the Centrality of Recycling

Recycling is the most prevalent CE practice across the UK, comprising 42.8% of all CE activities. This focus on recycling is consistent with the traditional emphasis on material recovery; however, it raises questions about whether the broader potential of CE is being fully exploited. The literature emphasizes that upstream practices, such as reduction and reuse, often deliver greater economic and environmental benefits by retaining more value within the economy and minimizing environmental impact [6, 66].

The UK also lags behind leading European peers in upstream emphasis. In the Netherlands, for example, ‘refuse’ and ‘reduce’ measures together account for approximately 35% of recorded CE actions [67], and the Dutch government has set a national target that all sectors halve primary raw-material use by 2030. Likewise, Finland’s 2022 Circular Economy Indicators report shows that nearly 40% o municipal and regional CE policies there prioritize reduction and reuse, well above the UK’s current upstream share [68]. This disparity suggests a risk of path-dependency if UK policy remains overly focused on downstream recycling, rather than shifting more resources toward upstream interventions that preserve greater economic and environmental value.

The emphasis on recycling varies regionally, reflecting both regional priorities and sectoral strengths. For instance, in Scotland, the Waste Management sector’s dominance in recycling activities is highlighted by projects like the Forth Replacement Crossing, where the old Forth Road Bridge was retained and repurposed. This project not only minimised material consumption but also demonstrated significant cost savings, aligning with the ReSOLVE framework’s “Reduce” and “Reuse” strategies promoted by the Ellen MacArthur Foundation (Arup, 2024). Similarly, the collaboration between Tata Steel and Arup on steel reuse showcases how recycling can generate economic savings of up to 27% for warehouses and up to 43% for offices, while also reducing the environmental footprint.

In Northern Ireland, the focus on the Emission & Net Zero sector, with 53.48% of CE activities directed towards recycling, reflects a strategic alignment with the region’s broader environmental goals. This is in line with Northern Ireland’s investments in renewable energy systems aimed at carbon reduction, which are crucial for meeting the UK’s 2050 net-zero targets. The emphasis on recycling within this sector, while significant, suggests potential for integrating more diverse CE strategies, such as those seen in Scotland’s Zero Waste Plan, which aims for a 70% recycling rate by 2025 while also promoting reduction and reuse.

England’s approach to CE, particularly in the Construction & Demolition sector, which accounts for 11.14% of CE activities, demonstrates a more balanced strategy. This sector shows a significant commitment to reduction, with 25% of its CE activities focused on reducing material use, exemplified by the Angel Building in Islington, London. This project retained the existing reinforced concrete frame, thereby reducing both embodied energy and construction costs. Such practices align with the recommendations in the “London’s CE Route Map,” which advocates for design adaptability and disassembly as critical elements of a sustainable construction strategy.

However, the relatively low involvement of sectors such as advanced manufacturing and engineering, which contribute less than 1% to CE activities in England, points to a missed opportunity. The “West Midlands CE Routemap” identifies the region’s potential to leverage Industry 4.0 technologies, such as real-time monitoring and predictive maintenance, to enhance CE practices within these sectors. By integrating these technologies, England could significantly boost its CE performance, particularly in high-tech industries that have traditionally lagged in CE adoption. To integrate advanced manufacturing and engineering into CE, targeted measures are needed. Incentivising IoT, enabled predictive maintenance and digital, twin tools can enable real, time asset tracking and component reuse [69]. A pilot fund for advanced remanufacturing technologies would allow SMEs to test circular processes on a small scale, assessing costs and material recovery before expanding. Simultaneously, industry-led training in data analytics and circular design will equip technicians to apply these innovations effectively, supporting rapid scaling once proven viable.

### Regional Disparities and Strategic Focus

The regional analysis reveals significant disparities in CE implementation across the UK, reflecting both the unique challenges and opportunities each region faces. Scotland and Northern Ireland’s strong focus on Emission & Net Zero initiatives, with 43.89% and 53.48% of their respective CE activities dedicated to these areas, underscores a strategic alignment with national decarbonization goals. Scotland’s ambitious targets, outlined in its “CE Waste Route Map to 2030,” include reducing landfill waste by 30% and achieving a 70% recycling rate by 2025. These targets are supported by infrastructure like the Glasgow Recycling and Renewable Energy Centre, which plays a critical role in the region’s waste management and resource recovery strategy.

Wales, on the other hand, exhibits a more traditional approach, with 46.07% of its CE activities focused on waste management. This suggests a reliance on downstream practices, which, while valuable, may not fully capture the potential of a CE. The Welsh Government’s “Beyond Recycling” strategy aims to shift this focus by targeting a 70% recycling rate by 2025, alongside a 26% reduction in waste generation. Initiatives such as the Ice Arena Wales in Cardiff, where steelwork was assessed for future reuse, exemplify the move towards more sustainable practices, but further emphasis on reduction and reuse could align Wales more closely with higher-value retention strategies advocated in the literature (Achterberg et al., 2016).

England’s CE practices reflect a more balanced approach, with significant contributions from various sectors and stakeholders. However, the underrepresentation of advanced manufacturing and engineering sectors points to a gap that could be addressed through targeted regional strategies. The “West Midlands CE Routemap” highlights the region’s potential to drive CE through low-carbon automotive technologies, supported by key players like Jaguar Land Rover. By focusing on reducing non-renewable resource use in manufacturing, as suggested in the Routemap, England could strengthen its CE practices and contribute more effectively to the UK’s overall CE goals.

In London, the “Our Waste, Our Resources” strategy emphasizes the importance of consistent recycling programs and waste reduction initiatives. London’s construction sector, responsible for 48% of the city’s waste, has adopted circular practices that include reducing waste through design for disassembly and adaptability. The Brighton Waste House, Europe’s first permanent public building made almost entirely from discarded materials, serves as a prime example of how innovative design can drive significant reductions in material use, aligning with the Reduce and Reuse strategies of the CE.

### Stakeholder Engagement: Challenges and Opportunities

Stakeholder engagement emerges as a critical factor in the successful implementation of CE practices across the UK. Local Authorities, Media, and the General Public are the most active participants, accounting for nearly half of all CE activities. This strong engagement is vital for driving public awareness and support for CE initiatives. For instance, the “CE Route Map for Glasgow 2020–2030” highlights how the Glasgow Chamber of Commerce has engaged over 650 businesses to adopt circular practices, underscoring the importance of local business engagement in the CE transition. To drive behavioural change, public engagement campaigns should leverage “nudge”strategies, such as social, norm messaging and default opt-in schemes for reusable containers, to harness media reach and encourage widespread adoption of circular actions.

However, there are significant disparities in stakeholder involvement across regions, with some areas, like Wales and Northern Ireland, showing limited participation from trade organizations and the private sector. This poses challenges to achieving a fully integrated CE strategy. The “Beyond Recycling” strategy by the Welsh Government addresses these gaps by fostering multi-stakeholder collaboration, with initiatives like Cardiff University’s RemakerSpace playing a pivotal role in driving innovation and community engagement. A targeted policy incentive, such as a tax credit for remanufacturing equipment investments, could further encourage trade organisations and businesses to participate by improving the financial case for circular operations.

Technological innovation is also becoming increasingly important in stakeholder engagement, particularly in managing complex waste streams such as electronic waste (e-waste). The “Electronic Waste and the CE” report by the House of Commons Environmental Audit Committee highlights the UK’s status as the second-largest producer of e-waste globally, generating 23.9 kg per capita annually. The report calls for stronger policies to enforce the right to repair and improve recycling methods, which could unlock significant economic value, up to $62.5 billion annually, from materials like gold, silver, and copper currently lost due to inefficient recycling practices.

The “West Midlands CE Routemap” also emphasizes the role of innovation in stakeholder engagement, particularly through the development of industrial symbiosis networks that facilitate the exchange and reuse of materials across sectors. By fostering such networks, the UK can enhance collaboration among stakeholders and promote more effective resource use, contributing to the overall success of the CE transition.

### Towards a Unified UK-Wide CE Roadmap

The development of a unified UK-wide CE roadmap is critical for harmonising the diverse approaches and addressing the regional disparities and sectoral gaps identified in this study. These regional disparities show that uniform policies are inadequate. A joint taskforce should align national strategy with regional priorities, support lagging areas, and scale successful local innovations to strengthen CE implementation. Such a roadmap must begin with the establishment of a clear and strategic vision, guided by time-bound objectives that reflect the varying levels of maturity across different regions and sectors. Drawing from the structured framework proposed by Abu-Bakar & Charnley (2024), this vision should align with the broader national goals of sustainability and economic resilience, while being flexible enough to adapt to regional strengths, such as Scotland’s focus on decarbonisation and Northern Ireland’s emphasis on Emission & Net Zero initiatives.

Governance is a cornerstone of this unified roadmap. It must involve establishing robust coordination mechanisms between national and regional authorities to ensure that local strategies are aligned with national CE objectives. This governance structure should clearly define roles and responsibilities, particularly for local authorities who are pivotal in implementing CE practices at the grassroots level. Effective governance will also require a collaborative approach, engaging key stakeholders such as businesses, local governments, and the public to drive CE initiatives. This is particularly important in regions like Wales and Northern Ireland, where private sector engagement has been relatively limited, yet is crucial for achieving a fully integrated CE strategy.

The roadmap must define precise Key Performance Indicators (KPIs) and metrics to measure progress. These KPIs should be aligned with the overarching goals of the CE, focusing not only on recycling but also on upstream practices such as reduction, reuse, and remanufacturing. The underrepresentation of sectors like advanced manufacturing and engineering, as highlighted in the findings, points to a need for targeted interventions. The roadmap should incorporate strategies to bolster these sectors, such as through the integration of Industry 4.0 technologies, which can enhance resource efficiency and foster innovation.

Action planning is another critical layer, where the roadmap must outline specific, actionable steps to achieve the defined goals. This includes identifying key focus areas, such as the construction and demolition sector in England, which has demonstrated potential through projects that emphasise material reduction and reuse. The roadmap should also prioritise regions where CE practices are less developed, ensuring that all areas contribute to and benefit from the CE transition.

Stakeholder engagement, as revealed in this study, must be broadened and deepened. The roadmap should foster an ecosystem of collaboration, encouraging partnerships across sectors and regions. This could involve creating sector-specific working groups, regular stakeholder forums, and public-private partnerships to drive CE initiatives. The emphasis on stakeholder engagement is critical, as it will ensure that CE practices are not only adopted but sustained over the long term.

To translate our findings into actionable steps, three key policy levers should be adopted. First, the government should introduce enhanced capital allowances for remanufacturing equipment. By allowing businesses to deduct a larger percentage of these investments against their taxable income in the first year, companies will be more inclined to purchase and deploy machinery needed to disassemble, refurbish or rebuild products. This incentive will reduce reliance on virgin materials, create new skilled jobs in remanufacturing, and promote local supply chains rather than importing finished components.

Second, extending the reduced VAT rate to include repair and refurbishment services would make extending product lifespans more financially attractive to both consumers and businesses. Lowering the cost of repairing household appliances, electronics or furniture encourages households to keep items in use rather than replace them, while also helping small repair shops and social enterprises to thrive. Over time, this measure would shift market demand toward durable, service-oriented business models, reducing material throughput and waste generation.

Third, establishing a Joint Circular Economy Unit, jointly overseen by UK Government and the devolved administrations, would ensure that regional strengths and priorities are aligned under a common national vision. This Unit would be responsible for setting coherent UK-wide targets, standardising data collection methods, and allocating innovation funding where it can have the greatest upstream impact. By pooling resources and expertise, the Unit would reduce duplication, enable best-practice sharing, and foster collaboration among local authorities, industry and research institutions, accelerating the transition toward a fully integrated circular economy.

Finally, the roadmap should incorporate continuous monitoring and evaluation mechanisms to track progress against the KPIs. This will allow for adaptive management, where strategies can be refined based on performance data and emerging trends. To accelerate upstream action, we also recommend (i) eco-design regulations requiring modularity and disassembly for key products (e.g. electronics, furniture), (ii) public‐procurement criteria that award contracts based on lifetime material intensity, and (iii) ring‐fenced innovation grants to support SME remanufacturing pilots. The importance of monitoring is underscored by the need to remain responsive to the dynamic nature of CE practices, particularly in a diverse economic landscape like the UK.

## Study Limitation

This study, while comprehensive in its scope, presents several intrinsic limitations. The use of NLP and NER to analyse 36 CE roadmaps is highly dependent on the quality and structure of the textual data. Variations in how CE practices are documented across different regions and sectors may have led to inconsistencies in data extraction and interpretation. Additionally, the reliance on a sample of publicly available documents means that the analysis might not fully capture the latest or unpublished CE initiatives, potentially missing innovative practices, patterns or emerging trends. The methodology also inherently prioritises quantity over depth, possibly overlooking nuanced regional or sector-specific insights that a more qualitative approach might reveal. Furthermore, the study’s focus on a predefined set of “R” principles may have limited its ability to identify novel CE strategies that fall outside these categories. Future research could address these limitations by incorporating mixed-method approaches and expanding the dataset to include more diverse and recent sources.

### Future Studies

Building on this study’s findings, future research should focus on the implementation and scaling of upstream CE practices such as reduction, reuse, and remanufacturing across different UK regions. Investigating the integration of these practices within existing industrial frameworks, particularly in sectors like advanced manufacturing, could provide valuable insights into overcoming current barriers. Additionally, research should explore the impact of emerging technologies, such as AI-driven predictive maintenance and IoT-enabled resource tracking, on enhancing the effectiveness of CE initiatives. Understanding the regional disparities in CE adoption and identifying best practices for fostering stakeholder engagement will also be essential. These studies can inform the development of more targeted and adaptive CE policies, supporting a unified and resilient transition to a CE in the UK.

## Conclusion

This study provides a thorough examination of CE practices across the United Kingdom, highlighting the varied approaches and significant gaps in the nation’s transition towards a sustainable, CE. A key finding is the dominant focus on recycling across regions, which, while important, underscores the critical need to prioritise upstream practices such as reduction and reuse. These practices are more effective in retaining value and mitigating environmental impacts. The study’s novel application of NER and NLP has allowed for a detailed, data-driven analysis, revealing regional disparities in CE implementation, particularly in the balance between recycling and upstream activities like reduction and reuse. We suggest establishing a national objective to shift emphasis away from a recycling-centric approach toward upstream strategies, such as reduction, reuse and remanufacturing, to enhance value retention and environmental impact.

Sectoral and regional analyses demonstrate that while regions like Scotland and Northern Ireland are making significant strides by aligning their CE practices with national decarbonisation goals, others, such as Wales, still rely heavily on traditional waste management methods. England’s balanced yet underutilised potential in advanced manufacturing and engineering presents both challenges and opportunities for advancing CE. The study also highlights the crucial role of stakeholders, local authorities, businesses, and the public, in driving CE initiatives, though there is a clear need for more comprehensive and inclusive stakeholder engagement, especially in sectors and regions where CE practices are less developed.

This study strongly advocates for the development of a unified UK-wide CE roadmap that leverages regional strengths and also prioritises upstream practices and remanufacturing. Such a roadmap should be built on a clear vision that reflects the diversity of the UK’s regions and the varying levels of CE maturity across sectors. Effective governance structures that enhance coordination between national and regional governments, alongside targeted strategies to increase participation from underrepresented sectors, are crucial for achieving a CE that is both sustainable and economically resilient.

## Data Availability

All datasets used in this research are in the public domain and available upon reasonable request to the corresponding author.
